# Using a Marginal Structural Model to Design a Theory-Based Mass Media Campaign

**DOI:** 10.1371/journal.pone.0158328

**Published:** 2016-07-21

**Authors:** Hiromu Nishiuchi, Masataka Taguri, Yoshiki Ishikawa

**Affiliations:** 1 Policy Alternatives Research Institute, The University of Tokyo, Bunkyo-ku, Tokyo, Japan; 2 Department of Biostatistics and Epidemiology, Graduate School of Medicine, Yokohama City University, Yokohama, Kanagawa, Japan; 3 Department of Health and Social Behavior, School of Public Health, The University of Tokyo, Bunkyo-ku, Tokyo, Japan; Iranian Institute for Health Sciences Research, ACECR, ISLAMIC REPUBLIC OF IRAN

## Abstract

**Background:**

The essential first step in the development of mass media health campaigns is to identify specific beliefs of the target audience. The challenge is to prioritize suitable beliefs derived from behavioral theory. The purpose of this study was to identify suitable beliefs to target in a mass media campaign to change behavior using a new method to estimate the possible effect size of a small set of beliefs.

**Methods:**

Data were drawn from the 2010 Japanese Young Female Smoker Survey (n = 500), conducted by the Japanese Ministry of Health, Labor and Welfare. Survey measures included intention to quit smoking, psychological beliefs (attitude, norms, and perceived control) based on the theory of planned behavior and socioeconomic status (age, education, household income, and marital status). To identify suitable candidate beliefs for a mass media health campaign, we estimated the possible effect size required to change the intention to quit smoking among the population of young Japanese women using the population attributable fraction from a marginal structural model.

**Results:**

Thirteen percent of study participants intended to quit smoking. The marginal structural model estimated a population attributable fraction of 47 psychological beliefs (21 attitudes, 6 norms, and 19 perceived controls) after controlling for socioeconomic status. The belief, “I could quit smoking if my husband or significant other recommended it” suggested a promising target for a mass media campaign (population attributable fraction = 0.12, 95% CI = 0.02–0.23). Messages targeting this belief could possibly improve intention rates by up to 12% among this population. The analysis also suggested the potential for regulatory action.

**Conclusions:**

This study proposed a method by which campaign planners can develop theory-based mass communication strategies to change health behaviors at the population level. This method might contribute to improving the quality of future mass health communication strategies and further research is needed.

## Introduction

Mass media health campaigns are effective at influencing health behavior at the population level [1]. A recent systematic review showed that this is particularly the case for reducing smoking and secondhand smoke [2]. However, such campaigns can also have unfavorable effects on behavior [3]. For example, in Japan, a national youth antidrug media campaign resulted in increasing marijuana initiation and the “Think. Don’t Smoke” campaign may have inadvertently promoted tobacco smoking [4–6]. Given their potential to influence a large number of people, mass media communication strategies need to be carefully developed.

An essential first step in developing such strategies is to identify which determinants of the targeted health behavior are changeable by communication and have the greatest likelihood of effecting behavioral change [7]. Although the mechanisms of behavioral change are usually complex and involve several interacting components [8], behavioral theories can provide a framework to help identify beliefs that can be addressed through persuasive communication. Evaluating whether such beliefs are changed by health campaigns can allow us to understand why and how mass media health campaigns succeed or fail and will contribute to the future development of communication strategies.

Research on behavioral theory over the past century has suggested a number of determinants of health behavior. Careful consideration of these determinants suggests that only a limited number need to be considered in predicting and explaining a substantial proportion of variance in any behavior in any population [7,9–10]. These theories suggest that behavior is driven by intention. Past meta-analyses of the relationship between intention and behavior in prospective studies reveal that intentions account for 28% of the variance in behavior [11]. The implementation intention [12] (i.e., “I intend to do X in situation Y”) appears to be the key determinant of actual behavior [11].

Past research reveals that the intention to perform the behavior is based on three belief categories: (1) attitudes, (2) norms, and (3) perceived control (or self-efficacy) [7] based on the theory of planned behavior developed by Fishbein and Ajzen and applied to understand various health behaviors [13]. These are the specific beliefs that serve as the targets for intervention efforts, as they ultimately determine intention and behavior. Therefore, it is necessary to identify which attitudinal, normative, or control beliefs are the best candidates to address through persuasive communication. Given the variety of beliefs, however, the challenge for the development of mass communication strategies is to identify a more limited set of beliefs to serve as the focus for intervention efforts.

The simple approaches used in previous studies to evaluate effect included Pearson’s correlation coefficient or a single regression coefficient [13]. However, these methods can lead to overestimation of the effect of the identified belief on change in intention, because mass media campaigns usually use one message to address diverse audiences, while one-to-one communication can use messages tailored to each person. The correlation or regression coefficient used in the earlier work suggests the possible effect size at a personal level. However, an effective message at a personal level may not translate into an effective mass media campaign because the people for whom this message is effective might be very few in number and others might not be convinced. Hornik and Woolf [14] therefore proposed that a cross-sectional survey could be used to identify target beliefs that meet the following criteria: 1) the belief should predict who will or will not engage in the target behavior; 2) there should be enough people who do not already hold the belief to make the intervention worthwhile; and 3) the belief should be changeable by persuasive communication. Assessment of the correlation coefficient or single regression coefficient is only a rough indicator of the first of these and Hornik and Woolf noted that we should also consider the population with unfavorable or without favorable beliefs. They recommended evaluation of the possible effect size using the product of the difference in the rate of behavioral intention between people with unfavorable and favorable beliefs and the proportion of people with the unfavorable belief in the whole of the target population. This can be calculated using a simple cross-tabulation and similar concept appears in what epidemiologists call population-attributable fraction (PAF); epidemiologists have also proposed a more accurate method to estimate PAF, which controls for various confounders [15, 16]. Having a belief or a behavioral intention is influenced by individual characteristics, such as age, sex, and socioeconomic status, such that the baseline characteristics of those with the belief in question are likely to differ from those without. We therefore need to control for these differences when identifying the target belief for mass media health campaigns, or be unable to distinguish a significant difference in behavioral intention related to a changeable belief, and an apparent difference related to socioeconomic status.

The purpose of this study was to estimate an indicator of a mass media campaign’s effect on behavioral intention among a small set of beliefs. In this study, we aim to improve upon Hornik and Woolf’s approach by controlling for confounders, and to predict the extent of a campaign’s effect, providing a standard error and confidence interval (CI)of the possible effect size.

## Methods

### Data Set

Data were drawn from the 2010 Japanese Young Female Smoker Survey, which called *20–30 Dai Kituen Josei No Segmentation Ni Kansuru Chousa* in Japanese [17]. This cross-sectional survey was conducted by the Ministry of Health, Labour and Welfare in Japan using an Internet-based research organization. Young females were the only demographic segment with increasing smoking prevalence in Japan, while other demographic segments showed decreased smoking prevalence; therefore, the government conducted a survey using careful formative evaluation research based on the theory of planned behavior. The Internet-based research organization has one of the largest research panels, with approximately 1,460,000 voluntarily registered subjects. After identifying age and smoking status of the registered subjects, 3,603 potential respondents, who were all female smokers aged 20–39 years, were randomly selected from the panel and invited via email to complete an Internet-based survey. Five hundred people completed the questionnaire between March 13 and 14, 2010, which was a response rate of 13.9%. Anonymous data were provided to the researchers.

Survey measures included implementation intention to quit smoking, psychological beliefs (attitude, norms, and perceived control) and baseline characteristics. Implementation intention to quit smoking was assessed as a dichotomous (yes/no) variable with the statement “Do you have a plan to quit smoking?”

### Psychological Beliefs

The 2010 Japanese Young Female Smoker Survey used measures on psychological variables developed through formative elicitation research [13], which was conducted with 11 smokers. In total, 21 beliefs on attitude, 6 on norm, and 19 on perceived control were assessed as dichotomous (yes/no) variables and Table 1 presents the measures for each psychological belief.

**Table 1:** Basic Characteristics of Study Participants.

	n	(%)
Total	500	
Age		
20–29	146	(29.2)
30–39	354	(70.8)
Education		
Highschool or less	206	(41.2)
Some college	162	(32.4)
College graduate	132	(26.4)
Employment Status		
Unemployed	166	(33.2)
Irregular employment	181	(36.2)
Regular employment	153	(30.6)
Household Income		
Did not respond	73	(14.6)
<4,000,000 JPY	196	(39.2)
4,000,000 to <8,000,000 JPY	178	(35.6)
≥ 8,000,000 JPY	53	(10.6)
Marrital Status		
Unmarried	265	(53.0)
Married	235	(47.0)
Intention to Quit Smoking		
Yes	67	(13.4)
No	433	(86.6)

### Baseline Characteristics

Characteristics measured were age (20–29, 30–39 years), education (less than high school, high school graduate, some college or technical school, and college graduate or more), household income (< 4 million JPY, 4 to <8 million JPY, and ≥8 million JPY), employment status (unemployment, irregular employment, or regular employment) and marital status (married or not married).

### Statistical Analysis

As mentioned previously, we can estimate the effect of a mass media campaign on behavioral intention using the product of 1) *ΔY*, the difference in the rate of behavioral intention between people with unfavorable and favorable beliefs; and 2) *p*, prevalence of unfavorable beliefs in the whole of the target population. *ΔY* × *p* is an indicator based on Hornik and Woolf’s [14] criteria, which is the numerator of what epidemiologists call PAF. We call this indicator population-attributability (PA).

Though we can estimate both *ΔY* and *p* easily from cross tabulation using cross-sectional data, they considered neither error nor confounders. Therefore, we proposed estimation of the standard error using the delta method under the assumption of (approximate) independence as follows:

$$SE\left(\Delta \hat{Y}\times \hat{p}\right)=\sqrt{\Delta {\hat{Y}}^{2}\times SE{\left(\hat{p}\right)}^{2}+{\hat{p}}^{2}\times SE{\left(\Delta \hat{Y}\right)}^{2}}$$

Moreover, we can obtain the 95% CI of the PA by calculating $\Delta \hat{Y}\times \hat{p}\pm 1.96SE\left(\Delta \hat{Y}\times \hat{p}\right)$ and p-value, given asymptotic normality.

In addition, we also proposed estimation of the adjusted $\Delta \hat{Y}$ controls for various confounders such as socioeconomic status with the marginal structural model (MSM) [18, 19]. The adjusted $\Delta \hat{Y}$ and the standard error can obtain the regression coefficient and the robust standard error of MSM weighted by propensity score estimated from confounders such as age, education, employment status, household income, and marital status. SAS software (v. 9.3; SAS Institute, Cary, NC) was used for all statistical analyses. The estimation details are shown in the Appendix.

### Ethical Considerations

The Japanese Ministry of Health, Labour and Welfare conducted the original survey using an Internet panel. Informed consent was obtained by filling out and submitting a Web form that we cannot access. Our research uses secondary analysis of anonymous data. The Office for Life Science Research Ethics and Safety, at the University of Tokyo approved our research (approval number: #15–1).

## Results

### Descriptive Data

In total, 13% of the study participants had an implementation intention to quit smoking. Twenty-nine percent of participants were 20–29 years old, and 71% were 30–39 years old. Table 1 shows the characteristics of the study participants.

### Population Attributable Fraction and Confidence Interval

Table 2 shows the PA for each belief obtained using Hornik and Woolf’s method [14]. The top five beliefs, by magnitude of PA, were: (1) I could quit smoking if my workplace became entirely smoke-free (PA = 0.27, 95% CI = 0.12–0.41); (2) I could quit smoking if places like transportation facilities, government offices, schools, and hospitals became entirely smoke-free (PA = 0.27, 95% CI = 0.06–0.47); (3) I could quit smoking if my close friends recommended it (PA = 0.23, 95% CI = 0.05–0.41); (4) I feel that it is foolish to ignore the warnings given to me about the dangers of smoking (PA = 0.17, 95% CI = 0.01–0.32; and (5) I could quit smoking if restaurants and amusement centers became entirely smoke-free (PA = 0.16, 95% CI = 0.04–0.28).

**Table 2:** Population-Attributability Using Hornik and Woolf’s Method.

Target Belief	Δ*Y*	*p*	PA	95%CI of PA			p-value	
Attitude								
Smoking is fun.	0.07	0.93	0.07	-0.06	-	0.19	0.306	
My smoking affects the health of those around me.	0.10	0.78	0.08	0.02	-	0.14	0.014	*
I like the look of people smoking.	-0.02	0.36	-0.01	-0.03	-	0.01	0.484	
If I became ill because of my smoking, it would upset people close to me.	0.11	0.70	0.08	0.02	-	0.13	0.004	*
I become more relaxed when I'm smoking.	0.03	0.98	0.03	-0.18	-	0.24	0.758	
People around me think I have no intention of quitting smoking, because I have not already done so.	-0.04	0.91	-0.04	-0.13	-	0.04	0.342	
I would become irritable and affect the people around me if I tried to quit smoking.	0.11	0.77	0.09	0.02	-	0.15	0.008	*
Smoking is bad for my health.	0.11	0.71	0.08	0.03	-	0.13	0.004	*
My friends and family prefer me to be happy and smoking rather than unhappy and quitting.	0.04	0.59	0.02	-0.01	-	0.06	0.236	
It bothers me that I can't help but smoke.	0.02	0.84	0.02	-0.05	-	0.09	0.593	
I can concentrate better and do more at my work while I'm smoking.	0.04	0.51	0.02	-0.01	-	0.05	0.155	
I think people think I am foolish because I ignore the harm caused by smoking.	0.12	0.80	0.10	0.03	-	0.17	0.008	*
I become less stressed and nervous when I am smoking.	0.02	0.80	0.02	-0.04	-	0.08	0.554	
People around me say I should stop smoking.	-0.02	0.93	-0.02	-0.12	-	0.09	0.760	
Smoking supports my decision making.	-0.05	0.91	-0.04	-0.13	-	0.04	0.307	
I feel that it is foolish to ignore warnings about the dangers of smoking.	0.18	0.94	0.17	0.01	-	0.32	0.038	*
The first time I smoke again after a break always tastes best.	-0.04	0.51	-0.02	-0.05	-	0.01	0.142	
I would be more active and energetic if I did not smoke.	0.11	0.90	0.10	-0.01	-	0.20	0.074	
Smoking helps me lose weight.	-0.01	0.89	-0.01	-0.09	-	0.08	0.874	
Smoking makes my skin and teeth bad.	0.04	0.93	0.03	-0.08	-	0.15	0.583	
Smoking makes it harder to conceive a baby.	-0.14	0.96	-0.13	-0.16	-	-0.10	<0.001	*
Norm								
Thinking you should quit smoking: Husband or significant other	0.10	0.63	0.06	0.02	-	0.10	0.004	*
Thinking you should quit smoking: Children	0.04	0.77	0.03	-0.03	-	0.09	0.275	
Thinking you should quit smoking: Parents	0.05	0.38	0.02	0.00	-	0.04	0.068	
Thinking you should quit smoking: Friends	0.07	0.71	0.05	0.00	-	0.10	0.052	
Thinking you should quit smoking: Medical personnel around me	0.07	0.89	0.06	-0.03	-	0.16	0.204	
Thinking you should quit smoking: Those around you who have successfully quit	0.06	0.87	0.05	-0.04	-	0.14	0.251	
Perceived control								
I could quit smoking if I got married.	0.06	0.83	0.05	-0.03	-	0.12	0.216	
I could quit smoking if I was trying to get pregnant.	-0.04	0.45	-0.02	-0.04	-	0.01	0.232	
I could quit smoking while pregnant.	-0.05	0.52	-0.03	-0.06	-	0.01	0.103	
I could quit smoking if I was in the right age-range to have children.	-0.04	0.57	-0.02	-0.06	-	0.01	0.194	
I could quit smoking if my husband or significant other recommended it.	0.08	0.84	0.07	-0.01	-	0.15	0.082	
I could quit smoking if my close friends recommended it.	0.24	0.95	0.23	0.05	-	0.41	0.014	*
I could quit smoking if everywhere around me was smoke-free.	0.11	0.85	0.10	0.01	-	0.18	0.029	*
I could quit smoking if the price of cigarettes went up.	0.02	0.45	0.01	-0.02	-	0.04	0.422	
I could quit smoking if my smoking caused those around me to become ill.	0.05	0.70	0.03	-0.02	-	0.08	0.183	
I could quit smoking if there was an easy way.	-0.05	0.55	-0.03	-0.06	-	0.00	0.086	
I could quit smoking if I could fine a way that meant I did not gain weight.	0.00	0.69	0.00	-0.04	-	0.05	0.978	
I could quit smoking if there was a silver bullet.	-0.02	0.48	-0.01	-0.04	-	0.02	0.436	
I could quit smoking if I found a way without any monetary cost.	-0.02	0.44	-0.01	-0.04	-	0.02	0.465	
I could quit smoking if my workplace became entirely smoke-free.	0.29	0.92	0.27	0.12	-	0.41	<0.001	*
I could quit smoking if places like transportation facilities, government offices, schools, and hospitals became entirely smoke-free.	0.28	0.96	0.27	0.06	-	0.47	0.012	*
I could quit smoking if restaurants and amusement centers became entirely smoke-free.	0.17	0.90	0.16	0.04	-	0.28	0.010	*
I could quit smoking if I became ill or unhealthy.	0.01	0.49	0.00	-0.03	-	0.03	0.855	
I could quit smoking if the results of a physical examination showed that I should.	0.05	0.60	0.03	-0.01	-	0.07	0.115	
I could quit smoking if a physician strongly recommended it.	0.01	0.65	0.01	-0.03	-	0.05	0.647	

*ΔY*: Crude difference of behavioral intention rates between those with and without unfavorable beliefs.

*p*: Prevalence of unfavorable beliefs.

PA, population-attributability: Increase in the rate of behavioral intention under the ideal condition, in which unfavorable beliefs would be completely removed in the population

* p-value < 0.05

Table 3 shows the PA for each belief using the MSM and controlling for socioeconomic status. The top five beliefs, by magnitude of PA, were: (1) I could quit smoking if places like transportation facilities, government offices, schools, and hospitals became entirely smoke-free (PA = 0.21, 95% CI = 0.01–0.41); (2) I could quit smoking if my workplace became entirely smoke-free (PA = 0.19, 95% CI = 0.04–0.33); (3) I could quit smoking if restaurants and amusement centers became entirely smoke-free (PA = 0.16, 95% CI = 0.01–0.31); (4) I could quit smoking if my husband or significant other recommended it (PA = 0.12, 95% CI = 0.02–0.23); and (5) I think people think I am foolish because I ignore the harm caused by smoking (PA = 0.11, 95% CI = 0.03–0.20).

**Table 3:** Population-Attributable Fraction Using the Marginal Structural Model.

Target Belief	*ΔY*	*p*	PA	95%CI of PA			p-value	
*Attitude*								
Smoking is fun.	0.05	0.93	0.05	-0.10	-	0.20	0.514	
My smoking affects the health of those around me.	0.09	0.78	0.07	0.01	-	0.13	0.027	*
I like the look of people smoking.	-0.02	0.36	-0.01	-0.03	-	0.02	0.509	
If I became ill because of my smoking, it would upset people close to me.	0.11	0.70	0.08	0.02	-	0.13	0.005	*
I become more relaxed when I'm smoking.	0.31	0.98	0.30	-0.19	-	0.79	0.230	
People around me think I have no intention of quitting smoking, because I have not already done so.	-0.08	0.91	-0.08	-0.15	-	0.00	0.037	*
I would become irritable and affect the people around me if I tried to quit smoking.	0.10	0.77	0.08	0.01	-	0.15	0.023	*
Smoking is bad for my health.	0.10	0.71	0.07	0.02	-	0.13	0.006	*
My friends and family prefer me to be happy and smoking rather than unhappy and quitting.	0.04	0.59	0.02	-0.02	-	0.06	0.259	
It bothers me that I can't help but smoke.	0.01	0.84	0.01	-0.06	-	0.08	0.770	
I can concentrate better and do more at my work while I'm smoking.	0.03	0.51	0.02	-0.01	-	0.05	0.270	
I think people think I am foolish because I ignore the harm caused by smoking.	0.14	0.80	0.11	0.03	-	0.20	0.008	*
I become less stressed and nervous when I am smoking.	0.02	0.80	0.02	-0.05	-	0.08	0.603	
People around me say I should stop smoking.	-0.02	0.93	-0.02	-0.13	-	0.08	0.676	
Smoking supports my decision making.	-0.07	0.91	-0.06	-0.13	-	0.00	0.066	
I feel that it is foolish to ignore warnings about the dangers of smoking.	0.18	0.94	0.17	-0.01	-	0.34	0.059	
The first time I smoke again after a break always tastes best.	-0.05	0.51	-0.03	-0.06	-	0.00	0.074	
I would be more active and energetic if I did not smoke.	0.08	0.90	0.07	-0.04	-	0.18	0.200	
Smoking helps me lose weight.	-0.02	0.89	-0.02	-0.10	-	0.07	0.686	
Smoking makes my skin and teeth bad.	0.02	0.93	0.01	-0.09	-	0.12	0.794	
Smoking makes it harder to conceive a baby.	-0.14	0.96	-0.13	-0.16	-	-0.10	<0.001	*
*Norm*								
Thinking you should quit smoking: Husband or significant other	0.07	0.63	0.05	0.00	-	0.10	0.063	
Thinking you should quit smoking: Children	-0.02	0.77	-0.02	-0.07	-	0.04	0.583	
Thinking you should quit smoking: Parents	0.05	0.38	0.02	0.00	-	0.04	0.117	
Thinking you should quit smoking: Friends	0.07	0.71	0.05	0.00	-	0.11	0.060	
Thinking you should quit smoking: Medical personnel around me	0.08	0.89	0.07	-0.05	-	0.20	0.235	
Thinking you should quit smoking: Those around you who have successfully quit	0.06	0.87	0.05	-0.05	-	0.14	0.316	
*Perceived control*								
I could quit smoking if I got married.	0.12	0.83	0.10	-0.09	-	0.29	0.301	
I could quit smoking if I was trying to get pregnant.	-0.03	0.45	-0.01	-0.04	-	0.01	0.350	
I could quit smoking while pregnant.	-0.06	0.52	-0.03	-0.06	-	0.00	0.029	*
I could quit smoking if I was in the right age-range to have children.	-0.05	0.57	-0.03	-0.06	-	0.01	0.134	
I could quit smoking if my husband or significant other recommended it.	0.15	0.84	0.12	0.02	-	0.23	0.022	*
I could quit smoking if my close friends recommended it.	0.19	0.95	0.18	-0.08	-	0.44	0.167	
I could quit smoking if everywhere around me was smoke-free.	0.07	0.85	0.06	-0.02	-	0.14	0.165	
I could quit smoking if the price of cigarettes went up.	0.05	0.45	0.02	-0.01	-	0.05	0.212	
I could quit smoking if my smoking caused those around me to become ill.	0.04	0.70	0.03	-0.02	-	0.08	0.237	
I could quit smoking if there was an easy way.	-0.05	0.55	-0.03	-0.06	-	0.01	0.096	
I could quit smoking if I could fine a way that meant I did not gain weight.	0.01	0.69	0.00	-0.05	-	0.05	0.892	
I could quit smoking if there was a silver bullet.	-0.01	0.48	0.00	-0.04	-	0.03	0.833	
I could quit smoking if I found a way without any monetary cost.	-0.01	0.44	0.00	-0.03	-	0.02	0.750	
I could quit smoking if my workplace became entirely smoke-free.	0.20	0.92	0.19	0.04	-	0.33	0.011	*
I could quit smoking if places like transportation facilities, government offices, schools, and hospitals became entirely smoke-free.	0.22	0.96	0.21	0.01	-	0.41	0.044	*
I could quit smoking if restaurants and amusement centers became entirely smoke-free.	0.18	0.90	0.16	0.01	-	0.31	0.034	*
I could quit smoking if I became ill or unhealthy.	0.00	0.49	0.00	-0.03	-	0.03	0.934	
I could quit smoking if the results of a physical examination showed that I should.	0.03	0.60	0.02	-0.02	-	0.06	0.300	
I could quit smoking if a physician strongly recommended it.	0.00	0.65	0.00	-0.04	-	0.04	0.975	

*ΔY*: Adjusted difference of behavioral intention rates between those with and without unfavorable beliefs, after controlling for socioeconomic status.

*p*: Prevalence of unfavorable beliefs.

PA, population -attributability: Increase in the rate of behavioral intention under the ideal condition, in which unfavorable beliefs would be completely removed in the population, after controlling for socioeconomic status

* p-value < 0.05

## Discussion

Not all mass media health campaigns have a positive impact on health behavior [3]. Therefore, it is important that campaign strategy development be based on behavioral theory to identify the critical beliefs that will play an important role in changing intention, rather than a more arbitrary development method [7]. Despite this, methods for selecting target beliefs for mass media health campaigns have received little attention since Hornik and Woolf [14].

The most important contribution of our study is to propose a new method, and demonstrate its use to identify a small set of critical beliefs that will lead to a greater possibility of changing behavioral intention. Our method, based on the MSM, identified slightly different target beliefs from Hornik and Woolf’s model, because we controlled for socioeconomic status. Though the differences between the two methods were very small in this study, the differences might be more important in other cases, for example, when designing mass media campaigns to target a more diverse population with more age groups, broader ethnicity and nationality or larger disparity of income and education. Though there were limited beliefs showing statistically significant PA by MSM, the belief “I could quit smoking if my husband or significant other recommended it” provides a reasonable primary target for a communication strategy, given the size of PA and the possibility of change by communication. Campaign planners could target young men, rather than young female smokers, to persuade them to advise their partners to stop smoking. This belief could not be identified using Hornik and Woolf’s method [14] because of confounding, and a mass media message targeting this belief is expected to achieve more behavioral change than other beliefs with less significant PAs. It is hoped that this method will help campaign planners to translate findings from behavioral research into daily practice. However, it is important to note that identifying the target belief does not necessarily lead to development of a suitable campaign message. Communication theory, rather than behavioral theory, should guide this aspect of campaign planning [7]. To develop effective communication strategies, further research will be required to clarify how messages that can change the identified target beliefs may be crafted.

The second important implication of our study is to identify critical beliefs that may be the target of regulatory policy. For example, the top three beliefs by magnitude of PA identified by MSM were “I could quit smoking if places like transportation facilities, government offices, schools, and hospitals became entirely smoke-free,” “I could quit smoking if my workplace became entirely smoke-free,” and “If restaurants and amusement centers became entirely smoke-free, I could quit smoking.” These aspects are not susceptible to persuasive communication, but could be changed by regulatory policy. Therefore, our method could inform not only mass media health campaign planners but also policy makers. The results also suggest the possibility of targeting mass media campaigns at administrators of public facilities, offices, and restaurants, rather than young female smokers.

This study has several limitations. First, we did not consider “priming,” which is an alternative communication strategy [7]. Hornik and Woolf [14] suggested that a majority of people should not hold the target belief for persuasive communication, but “priming” or enforcing existing beliefs held by a majority may be a suitable alternative strategy for communication design. It could be argued that emphasizing existing beliefs related to behavioral intention would increase the likelihood that they would be considered. Future research will be required to develop and examine methods to estimate the effect size and its CI by “priming” the target beliefs. Second, the estimated effect size may not be very close to the causal effect size, because the propensity score method cannot control for unknown or unmeasured confounders. The data set used in this study had very limited items thought to be potential confounders; therefore, and there might be others that were not identified. Although outside the scope of our research, using sensitivity analyses for unmeasured confounding [20] would help campaign planners understand the limitations of the estimation in effect size. Third, the data set only includes young Japanese female participants who could respond to Internet-based surveys. This might be biased population and our result could not be applicable to other population. Fourth, our proposed method did not consider the equality point of view. Changing the target belief at population level through a mass media health campaign might be counterproductive for a specific subgroup. Some researchers have argued that the population approach can be detrimental for vulnerable groups [21]; therefore, future research should explore and develop methods to identify suitable beliefs leading to a greater likelihood of reducing disparities in behavioral intention.

In summary, this study proposed and simulated a method to identify limited sets of theory-based beliefs to serve as targets for persuasive communication in a mass media health campaign. We hope this method will help to improve the quality of mass communication strategies by improving targeting.

## Appendix

Let *A* be the indicator variable of an exposure or unfavorable belief such as “*Smoking is fun*” and *Y* be an observed dichotomous outcome or behavioral intention. *Y* = 1 in this study means that the subject intends to quit smoking. *X* represents multicategory confounding factors such as age, education, employment status, household income, and marital status. Using the potential outcome model [22], *Y*_*a*_ (*a* = 0,1) represents the counterfactual outcomes that would have been observed under exposure category *a*. Our objective is to estimate the possible reduction in the probability of the outcome under the ideal condition, in which any unfavorable belief obstructing the behavioral intention is completely removed. Using a formula, we wish to estimate population-attributability (PA) as follows:

$$\begin{array}{l} \text{Pr}(Y=1)-\text{Pr}(Y_{0}=1)\\ =\sum_{a=0}^{1}\left\{\text{Pr}(Y_{a}=1|A=a)-\text{Pr}(Y_{0}=1|A=a)\}\text{Pr}(A=a\right)\\ =\{\text{Pr}(Y_{1}=1|A=1)-\text{Pr}(Y_{0}=1|A=1)\}\text{Pr}(A=1). \end{array}$$ (1)

The last expression of Eq 1 is the product of the causal risk difference in the exposed group and the exposure probability. Unfortunately, Pr(*Y*_0_ = 1 | *A* = 1) in Eq 1 is not estimable without assumptions because *Y*_0_ is not observed in the exposed group. If exposure *A* is randomized effectively, we can expect the following *unconditional exchangeability* assumption to hold [23]: *Y*_*a*_∐*A*. Under this assumption and the usual consistency assumption, which means *Y*_*A*_ = *Y*, Eq 1 can be re-expressed as:

{Pr(Y=1|A=1)−Pr(Y=1|A=0)}Pr(A=1). (2)

Expression (2) is the product of the unadjusted risk difference and the exposure probability, which we called Δ*Y* and *p* in the body text. By replacing the probabilities in Eq 2 by sample proportions, we obtain an estimator of Eq 2, which is identical to that proposed by Hornik and Woolf [14].

However, in a cross-sectional survey, the assumption of *unconditional exchangeability* may not apply because of confounding. Instead, we make the following *conditional exchangeability* assumption [19]: *Y*_*a*_∐*A*|*X*. This implies that there is no unmeasured confounding within levels of the measured confounders. Under this assumption, we can estimate the causal risk difference (in the exposed group) in (1) by using the marginal structural model (MSM) [18, 19]. This involves three steps:

1. Fit the regression model for the association of confounder–exposure relationship. For instance, fit the following logistic regression model:
  $$\text{logit}\{\text{Pr}(A=1|X=x)\}=\alpha_{0}+\alpha_{1}^{}x,$$ (3)
  using standard software such as SAS (SAS Institute, Cary, NC).
2. Using the predictive value of the fitted regression model (3), calculate weights $w_{i}=\frac{\text{Pr}(A=1|X=x_{i})}{\text{Pr}(A=0|X=x_{i})}$ for each individual *i* in the unexposed group (*A* = 0). *w*_*i*_ = 1 for the exposed group (*A* = 1).
3. Estimate causal parameters of the following MSM:
  $$\text{Pr}(Y_{a}=1|A=1)=\beta_{0}+\beta_{1}a,$$ (4)
  by fitting the association model Pr(*Y* = 1 | *A* = *a*) = *γ*_0_ + *γ*_1_*a* with weights *w*_*i*_.

From Eq 4, the proposed estimator of Eq 1 is obtained by:

$${\hat{\beta }}_{\text{1}}\times \hat{\text{P}}\text{r}(A=1).$$ (5)

Similar estimators have been proposed in the context of estimation of the population attributable fraction (PAF) [15–16]. The PAF proposed in the earlier study could be presented as:

$$\frac{{\hat{\beta }}_{1}\times \hat{\text{P}}\text{r}\left(A=1\right)}{\hat{\text{P}}\text{r}\left(Y=1\right)}$$ (6)

To estimate the confidence interval (CI) of the PA, we assumed (approximate) independence between ${\hat{\beta }}_{\text{1}}$ and $\hat{\text{P}}\text{r}(A=1)$. ${\hat{\beta }}_{\text{1}}$ and the robust standard error $SE({\hat{\beta }}_{\text{1}})$ were estimated with SAS’s GENMOD procedure using the weight *w*_*i*_ calculated by the logistic procedure. $\hat{P}r\text{(}A=1)$ and the standard error can be estimated easily from the data step. Under the assumption of independence and based on the delta method, the standard error of the proposed estimator is

$$SE\text{(}{\hat{\beta }}_{\text{1}}\times \hat{\text{P}}\text{r}(A=\text{1))}=\sqrt{{\hat{\beta }}_{\text{1}}{}^{\text{2}}\times SE{(\hat{\text{P}}\text{r}(A=\text{1))}}^{\text{2}}+\hat{\text{P}}\text{r}(A={\text{1)}}^{\text{2}}\times SE{\left({\hat{\beta }}_{\text{1}}\right)}^{\text{2}}}.$$ (7)

Given asymptotic normality, we can obtain the 95% CI of ${\hat{\beta }}_{\text{1}}\times \hat{\text{P}}\text{r}(A=1)$ by calculating ${\hat{\beta }}_{\text{1}}\times \hat{\text{P}}\text{r}(A=1)$±1.96 SE, and the p-value. This also applies to Hornik and Woolf’s simple cross-tabulation. SAS v.9.3 (SAS Institute, Cary, NC) was used for all statistical analyses.
